# Immunogenicity and Safety of a Quadrivalent Influenza Vaccine in Population Aged 3 Years and Older in Chile and the Philippines: A Phase 3, Non-Inferiority, Double-Blind, Randomized Controlled Clinical Trial

**DOI:** 10.3390/vaccines12080892

**Published:** 2024-08-07

**Authors:** Wanqi Yang, Pablo A. González, Qianqian Xin, Mari Rose De Los Reyes, Ralph Elvi Villalobos, Charissa Fay Corazon Borja-Tabora, Nancy Nazaire Bermal, Alexis M. Kalergis, Dan Yu, Wenbin Wu, Susan M. Bueno, Liqun Huo, Mario Calvo, QINF Study Group, Gang Zeng, Jing Li

**Affiliations:** 1 Sinovac Biotech Co., Ltd., Beijing 100085, China; 2 Millennium Institute on Immunology and Immunotherapy, Santiago 7810128, Chile; 3 Facultad de Ciencias Biológicas, Pontificia Universidad Católica de Chile, Santiago 7810128, Chile; 4 Sinovac Life Sciences Co., Ltd., Beijing 102601, China; 5 Las Pinas Doctors Hospital, Metro Manila 1008, Philippines; 6 Philippines General Hospital, Metro Manila 1000, Philippines; 7 Tropical Disease Foundation, Inc., Metro Manila 1230, Philippines; 8 San Juan De Dios Hospital, Pasay 1300, Philippines; 9 Departamento de Endocrinología, Facultad de Medicina, Pontificia Universidad Católica de Chile, Santiago 7810128, Chile; 10 Institute of Medicine, Universidad Austral de Chile, Valdivia 5090000, Chile

**Keywords:** quadrivalent influenza vaccine, non-inferiority, immunogenicity, safety

## Abstract

Objectives: In this study, we aimed to evaluate the non-inferiority of a quadrivalent influenza vaccine (QIV) developed by Sinovac Biotech Co., Ltd. (Sinovac, Beijing, China) by comparing its immunogenicity and safety with a comparator QIV (Vaxigrip Tetra^®^) in a population aged 3 years and older in Chile and the Philippines. Methods: A phase 3, non-inferiority, double-blind, randomized controlled, multicenter clinical trial was conducted in the southern hemisphere (SH) 2023 influenza season. Participants aged ≥ 3 years old with stable health were randomized 1:1 to receive either Sinovac QIV or comparator QIV. The co-primary outcomes were immunological non-inferiority for Sinovac QIV versus the comparator against each strain contained in the vaccines in terms of seroconversion rates (SCRs) and geometric mean titers (GMTs) of hemagglutination inhibition (HI) antibodies 28 days after final vaccination. Results: A total of 2039 participants were vaccinated (1019 Sinovac QIV; 1020 comparator QIV). Sinovac QIV induced non-inferior immune responses to all four strains as compared to comparator QIV, with slightly higher GMTs than those of comparator QIV: GMT ratios (lower limit 95% confidence interval (CI)) were 1.8 (1.6) for A(H1N1), 1.4 (1.3) for A (H3N2), 1.3 (1.1) for B Victoria and 1.2 (1.1) for B Yamagata; observed seroconversion rate differences (lower limit 95% CI) were 9.6% (6.7) for A(H1N1), 7.0% (3.5) for A(H3N2), 2.4% (−0.03) for B Victoria and 6.8% (3.0) for B Yamagata. Adverse reactions were similar across the two groups and no vaccine-related serious adverse events were reported. Conclusions: The immunogenicity of Sinovac QIV was non-inferior to that of the comparator QIV in these populations aged 3 years and older, and safety was comparable.

## 1. Introduction

Influenza is a highly contagious respiratory disease, caused by influenza A and B viruses circulating worldwide, which leads to significant morbidity and mortality annually [1]. Vaccination is considered the most effective way to prevent influenza [1]. 

Vaccination coverage varies across countries as affected by different factors, such as national immunization programs, recommendations from healthcare providers, low perceived need for vaccination, out-of-pocket costs, and lack of confidence in vaccines, among others [2,3]. 

Both Chile and the Philippines use influenza vaccines targeted for the southern hemisphere (SH) [4]. In Chile, influenza vaccines are often purchased by governmental funding and provided free of charge for individuals at high risk of developing severe complications [5]. In 2022, vaccination coverage was 88% in Chile for priority groups, such as healthcare personnel, pregnant women, older adults and children before peak influenza activity, and influenza vaccines were 49% effective in preventing hospitalizations during the predominantly A/H3N2 season [6]. 

The Philippines is considered a developing country with underdeveloped public healthcare systems, and the burden of influenza remains high at present [7]. Influenza vaccines were not included in the national immunization program in the Philippines, but they are accessible upon out-of-pocket payment [8]. Although many efforts have been made by the Philippines government, low vaccination rates remain a recurring problem, and only around 30% of Filipinos are aware of the influenza vaccine [7,8]. A recent study reported that the annual estimated influenza-attributable excess mortality rate in the Philippines was 5.09 per 100,000 individuals from 2005 to 2015 [9]. Therefore, increased vaccination is crucial for adequate protection of the population in the context of influenza seasons and potential future pandemics. 

An inactivated quadrivalent influenza vaccine developed by Sinovac Biotech Co., Ltd. (Sinovac QIV, Beijing, China) has been approved for preventing influenza-related disease in several countries. The reliable immunogenicity and safety of Sinovac QIV has been previously demonstrated in the Chinese population in previous studies during the Northern Hemisphere (NH) influenza seasons [10,11,12]. However, there are no study data available for this vaccine in diverse populations for the SH influenza season. Thus, we conducted a phase 3 clinical trial to assess the immunogenicity and safety of Sinovac QIV compared to the widely used quadrivalent influenza vaccine, Vaxigrip Tetra^®^, in populations aged 3 years and older in Chile and the Philippines during the SH 2023 influenza season.

## 2. Methods

### 2.1. Study Design and Population

This phase 3, randomized, double-blind, controlled, multicenter and non-inferiority clinical trial was conducted to assess the immunogenicity and safety of Sinovac-QIV compared to a licensed QIV (Vaxigrip Tetra^®^) in a population aged 3 years and older. The study was conducted in 9 study sites in Chile and the Philippines in the SH 2023 influenza season (Clinical trial registration: Identifier No. NCT05431725). 

Individuals aged 3 years and older with stable health were considered for inclusion. The key exclusion criteria included if they had received any influenza vaccine within the past 6 months; history of Guillain–Barré syndrome within 6 weeks after receiving any influenza vaccine in the past; history of allergies to any vaccine or vaccine ingredients; history of autoimmune diseases or immunodeficiency, or any immunosuppressant therapy within 6 months; or received blood products within 3 months, live attenuated vaccines within 14 days or subunit/inactivated vaccines within 7 days. Women were excluded if they were pregnant, breast-feeding or had pregnancy plans during the study. More detailed eligibility criteria can be found on ClinicalTrials.gov.

Participants who were 3–8 years of age and had prior receipts of ≥2 doses of influenza vaccine at least 4 weeks apart during the previous influenza season or who were ≥9 years old were considered “vaccine-primed”. Participants who were 3–8 years of age and had prior receipt of <2 doses of influenza vaccine during the previous influenza season were considered “vaccine-unprimed”. Vaccine-primed participants received one dose of study vaccine; vaccine-unprimed participants received two doses of study vaccines at an interval of 28 days. The study protocol and informed consent forms were approved by Institutional Ethics Committees of each site, and the study was conducted in compliance with the International Conference on Harmonization guidelines for Good Clinical Practice, Declaration of Helsinki and regulatory requirements of local authorities. Written informed consent was obtained from participants or children’s legal guardians prior to enrollment; adolescents aged 9–17 years also signed a written assent.

### 2.2. Study Vaccines

Study vaccines included QIV manufactured by Sinovac Biotech Co., Ltd. (Beijing, China), and a licensed comparator QIV (Vaxigrip Tetra^®^, Sanofi Pasteur, Lyon, France), both of which are inactivated, split-virion vaccines. Each dose (0.5 mL) of study vaccines contained 15 μg hemagglutinin (HA) of each of the four strains of influenza as recommended by World Health Organization (WHO) for the SH influenza season of 2023: A/Sydney/5/2021(H1N1)pdm09-like virus, A/Darwin/9/2021(H3N2)-like virus, B/Austria/1359417/2021(BV)-like virus, B/Phuket/3073/2013(BY)-like virus [13]. Eligible participants were administered either the study vaccine or the comparator intramuscularly at the lateral deltoid muscle of the upper arm. 

### 2.3. Randomization and Blinding

Participants were randomized in a ratio of 1:1 to receive Sinovac QIV or comparator QIV. They were randomized using the specified block method with stratification by age group (3–8 years, 9–17 years, 18–64 years, and ≥65 years) and country (Chile and the Philippines) and were assigned treatment groups using an interactive web response system (IWRS). All investigational vaccines were repackaged with blind labels. All participants, investigators and laboratory staff were blinded to vaccine allocation.

### 2.4. Immunogenicity Assessment 

Blood samples were collected before vaccination (day 0) and 28 days after final vaccination (day 28 for vaccine-primed participants and day 56 for vaccine-unprimed participants). Hemagglutination inhibition (HI) assay was used for testing the antibody titers by PPC laboratory (Protech Pharmaservices Corp., Taipei, Taiwan). Non-specific inhibitors were removed from serum samples by receptor destroying enzymes to avoid competitive non-specific binding. Serum samples were diluted and incubated with an influenza virus solution with 4 hemagglutination units/25 μL. The lower limit of antibody titer was 1:10, and values below this limit were imputed as 1:10. Turkey red blood cells were added to observe the highest serum dilution at which hemagglutination was absolutely inhibited. 

The primary objective of the study was to demonstrate the immunological non-inferiority of the Sinovac QIV versus the comparator QIV, as evaluated by seroconversion rate (SCR) and geometric mean titer (GMT). SCR was defined as the proportion of participants with a change in HI titer from <1:10 to ≥1:40, or a four-fold increase of HI titer if baseline HI antibody titers were ≥1:10. The secondary immunogenicity objective was to describe immune responses, as indicated by seroprotection rate (SPR) and geometric mean increase (GMI). SPR was defined as the percentage of participants with HI titer ≥ 1:40. Antibody responses were also evaluated based on Center for Biologics Evaluation and Research (CBER) criteria (lower limit of 95% confidence interval (CI) of SCR ≥ 40%, SPR ≥ 70% for individuals aged 3–64 years old; lower limit of 95% CI of SCR ≥ 30%, SPR ≥ 60% for the elderly aged ≥ 65 years old), and Committee for Medicinal Products for Human Use (CHMP) criteria (GMI value > 2.5 for 3–64 years and >2.0 for 65 years and older) [14,15].

### 2.5. Safety Assessment

Participants were monitored for 30 min after vaccination for immediate adverse reactions. Diary cards were used for collection of solicited local and systemic adverse events within 7 days of vaccination, while unsolicited adverse events and serious adverse events were collected for 28 days after each dose. Solicited local symptoms included pain, induration, swelling, erythema, rash and pruritus; solicited systemic symptoms included headache, cough, fatigue, fever, muscle pain, diarrhea, anorexia, vomiting, nausea, acute allergic reaction, skin and mucosa abnormality. Severity of adverse events was graded based on guidelines issued by the National Medical Products Administration, China (2019) [16]. Causality of adverse events and vaccination was determined by the study investigators.

### 2.6. Sample Size Determination and Statistical Analysis

The sample size was calculated to provide at least 80% power to demonstrate the non-inferiority for all 8 co-primary endpoints of SCRs and GMTs for each of the four strains in the overall population aged 3 years and older. According to SCR, 659 participants in each group were to be enrolled with the non-inferiority margin of −10% for SCR difference, one-sided alpha level of 0.025, and 60% SCR for the comparator QIV group. According to GMT, 760 participants in each group were necessary, given a non-inferiority margin of 2/3 for adjusted GMT ratio, a one-sided alpha level of 0.025, and a coefficient of variation of 0.8. Assuming a 20% drop-out rate, 1000 participants in each group should be enrolled based on the above maximum sample size.

SAS software version 9.4 (SAS Institute, Inc., Cary, NC, USA) was used for statistical analysis. Full analysis set (FAS) was used for analysis of demographic characteristics, which included all participants who were vaccinated with at least one dose of vaccine and had valid immunogenicity data before and after vaccination. Per-protocol set (PPS) was used to evaluate immunogenicity, which included all randomized participants who completed vaccination, had available HI antibody results, and did not have other factors affecting immunogenicity assessment. Safety set (SS) was used for safety evaluation, which included all vaccinated participants.

Immunogenicity non-inferiority was assessed for each vaccine strain by using SCRs and GMTs as co-primary endpoints. Immunogenicity non-inferiority was demonstrated if the lower bound of two-sided 95% CI for SCR difference (Sinovac QIV group minus comparator QIV group) ≥ −10%, and adjusted GMT ratio (Sinovac QIV group/comparator QIV group) ≥ 2/3 for each of the four antigen strains. The SCR and SPR against each strain were calculated, and their 95% CI were calculated by Clopper–Pearson method. The GMT and GMI of HI antibodies against each strain were calculated by geometric mean and 95% CI. GMTs were adjusted by using an analysis of covariance model fitted on log10 transformed post-vaccination HI titer, including the treatment group and age group as the fixed effect and baseline as covariate. The safety profile was described by counts and the proportion of participants reporting adverse events. Chi-square test or Fisher exact probability test was used to compare group difference for binary variables. *t* test or Wilcoxon rank-sum test was used to compare group difference for continuous variables.

## 3. Results

### 3.1. Study Population

**Figure 1:**
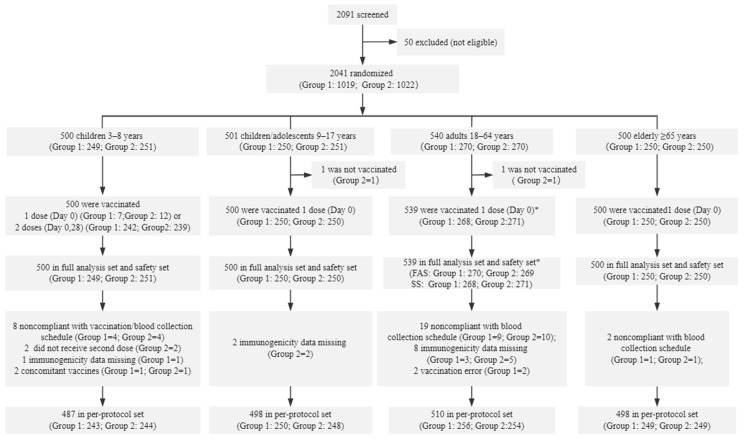
Screening, enrolment, and follow-up of participants. Abbreviations: Group 1= Sinovac QIV group; Group 2 = comparator QIV group; SS: safety set; FAS: full analysis set. * Two participants in 18–64 years group were randomized to the Sinovac QIV group, but mistakenly received the comparator QIV. They were analyzed in full analysis set of Sinovac QIV group, safety set of comparator QIV group, and excluded from per-protocol set. Blood collection schedule was Days 0 and 28 for participants who received 1 dose, and Days 0 and 56 for participants who received 2 doses.

Between April 2023 and July 2023, a total of 2041 participants were enrolled, including 334 in Chile and 1707 in the Philippines. 2039 participants received at least one dose of the study vaccines and were included for safety assessment (1017 in Sinovac QIV group and 1022 in comparator QIV group), including 500 (24.5%) participants aged 3–8 years, 500 (24.5%) aged 9–17 years, 539 (26.4%) aged 18–64 years and 500 (24.5%) aged 65 years and older. Among all vaccinated participants, 483 children aged 3–8 years were vaccinated for the first time and 481 of them received a second dose (242 in Sinovac QIV group and 239 in comparator QIV group). 1993 participants were included PPS for immunogenicity evaluation (998 in Sinovac QIV group and 995 in comparator QIV group) (Figure 1). 

The demographic characteristics of the participants were well balanced between treatment groups in terms of gender, age, height, weight, race and ethnicity upon full set analysis (Table 1). The mean age was 31.3 years (SD 25.7) in the Sinovac QIV group and 31.9 years (SD 25.9) in the comparator QIV group, respectively. Female, Asian and Not Hispanic or Latino comprised the majority of participants in the overall study population (Table 1).

### 3.2. Immunogenicity

Before vaccination, the SPRs of the HI antibody varied between 16.5% and 76.9% and GMTs were 10.0 to 64.8 against all four influenza strains contained in the vaccines. The baseline antibody levels were similar between the two groups in overall population (Supplement Table S1). The SPR of B Victoria is slighter higher in Sinovac QIV group than the comparator group in adolescents aged 9–17 years (31.2% vs. 21.8%, *p* = 0.0172); there’s no statistically significant difference in pre-vaccination antibody titers between two groups in other age strata (Supplement Table S1).

For primary endpoints, Sinovac QIV met non-inferiority criteria against each of the four influenza strains as compared to the comparator QIV 28 days after final vaccination in the overall study population. The SCR differences (Sinovac QIV minus comparator QIV) were 9.6% (95% CI: 6.7–12.5) for A/H1N1, 7.0% (95%CI: 3.5–10.5) for A/H3N2, 2.4% (95%CI: −0.03–4.9) for B Victoria, and 6.8% (95%CI: 3.0–10.7) for B Yamagata. The GMT ratios of Sinovac-QIV/comparator QIV were 1.8 (95%CI: 1.6–2.0) for A/H1N1, 1.4 (95%CI: 1.3–1.6) for A/H3N2, 1.3 (95%CI: 1.1–1.4) for B Victoria, and 1.2 (95%CI: 1.1–1.2) for B Yamagata (Table 2). In addition, except for the SCR of B Victoria, the SCRs and GMTs against all four strains of influenza in the Sinovac QIV group were higher than those in the comparator group, with significant differences (Table 3).

Upon analysis by age groups, Sinovac QIV elicited a robust immune response in each age stratum (3–8/9–17/18–64/65 years and older). The immune responses were non-inferior for Sinovac QIV versus comparator QIV in each age group, and met the CBER criteria for SCR and SPR, and also met the CHMP criteria for GMI (Table 2 and Table 3). At 28 days after final vaccination, the lower limit 95% CI of SCRs were 60.0% to 98.5% for Sinovac QIV recipients, and 49.4% to 98.5% for comparator QIV receipts in each age group, with the GMIs ranging from 4.0 to 20.8 and 3.8 to 17.6, respectively. The post-vaccination GMTs against all strains in the Sinovac QIV group were slightly higher than those in the comparator QIV group with statistical significance (except for B Yamagata in 3–8 years and ≥65 years, and B Victoria in 18–64 years) (Table 2 and Table 3). 

### 3.3. Safety

A total of 2039 participants received at least one dose of Sinovac QIV or comparator QIV. Adverse reactions occurred in 25.5% (259/1017) of Sinovac QIV recipients, and 27.0% (276/1022) of comparator QIV recipients, respectively (*p* = 0.4297), within 28 days after vaccination. Most adverse reactions occurred within 7 days post-vaccination, and were mild or moderate in severity. Only 10 (1.0%) of 1017 participants in Sinovac QIV group and 7 (0.7%) of 1022 in the comparator QIV group reported grade 3 adverse reactions, with the most common symptoms consisting of fever and vaccination site pain (Table 4). 

Among all participants, solicited local adverse reactions were reported by 16.3% (166/1017) in the Sinovac QIV group and 17.1% (175/1022) in the comparator QIV group (*p* = 0.6281), and vaccination site pain was the most common reported local symptom (15.4% for Sinovac QIV vs. 16.1% for comparator QIV). 152 (14.9%) of 1017 participants in the Sinovac QIV group and 163 (15.9%) of 1022 participants in the comparator QIV group reported solicited systemic adverse reactions. The most commonly reported solicited systemic symptom was headache (7.6% vs. 7.9% for Sinovac QIV and comparator QIV, respectively), followed by fever (5.0% vs. 5.6% for Sinovac QIV and comparator QIV, respectively) and fatigue (4.9% vs. 5.0% for Sinovac QIV and comparator QIV, respectively). Except for a slightly higher rate of pruritus at the of site vaccination in the comparator QIV group (*p* = 0.0077), there were no significant differences in the incidences of other solicited reactions between the two groups. Only 2.2% (22/1017 for Sinovac QIV) and 2.9% (30/1022 for comparator QIV) of participants in the two groups reported unsolicited adverse reactions, without significant difference (*p* = 0.2688) (Table 4).

During the study period, only two participants reported serious adverse events, with a cerebral infarction in Sinovac-QIV group and a basal ganglia hemorrhage in the comparator QIV group. Both events were assessed as not related to vaccination.

## 4. Discussion

The present phase 3, double blind, randomized control study is the first to report the immunogenicity and safety of Sinovac QIV outside China, with sites in Chile and the Philippines. The results of this study have demonstrated that the immune responses induced by Sinovac QIV were not inferior to, and even slightly higher than those of a widely used comparator QIV in individuals aged 3 years and older. In addition, Sinovac QIV fulfilled the CBER and CHMP criteria for immunogenicity evaluation in individuals aged 3–64 years and ≥65 years [14,15]. Immunogenicity profiles were satisfactory in the Chinese population aged 3 years and older in a previous phase 3 clinical trial [10]. The results from both studies support the use of Sinovac QIV in different populations in the northern and southern hemispheres.

In this study, over 95% of children aged 3–8 years were vaccine-unprimed and received two doses of QIV, which differs with participants of this age group receiving one dose of the vaccine in our previous phase 3 study in China [10]. The two-dose regimen notably increased the immune responses for SCR against all four strains, especially for B Victoria (100% vs. 55%) and B Yamagata (89% vs. 50%). Several studies have reported that the two-dose regimen is more effective against influenza compared with one dose [17,18]. Our data provides additional evidence for the use of a two-dose immunization schedule for influenza vaccines in children under 9 years old. 

In post hoc analysis of participants aged 18–64 years between two countries, higher immune responses were observed in participants from the Philippines, as compared to those from Chile (Supplementary Table S2). The lower immune responses in the Chilean population may be attributed to pre-existing antibodies after repeated vaccination. It has been reported pre-existing immunity resulted from repeated influenza vaccination could limit the updating of immune responses against new influenza viruses [19]. 

Noteworthily, the elderly have an increased risk of influenza-associated hospitalizations and risk of death compared with the younger population [1]. Thus, vaccination for preventing influenza in the elderly is important. A 2018 Cochrane review found that influenza vaccination had an effectiveness of 58% against influenza in elderly people aged 65 years and older [20]. In this study, although the elderly showed a slightly lower level of immune responses than children/adolescents, the responses in this age group were similar to those in adults and exceeded the CBER and CHMP criteria. 

The results suggest that QIV can elicit robust immune responses in the elderly to provide protection against influenza. Importantly, the safety profile of Sinovac QIV was similar to the comparator QIV. The incidences of solicited local and systemic reactions and unsolicited adverse reactions were similar among both groups. Most adverse reactions were mild or moderate. Vaccination site pain was the most reported local symptom, while headache, fever and fatigue were the most reported systemic symptoms. No vaccine related serious adverse event was observed. The results were similar to those obtained in our study in a Chinese population aged 3 years and older, as well as findings reported for other QIV [10,21,22].

The strengths of this study are the following. First, the large sample size enabled a meaningful comparison of the immunogenicity outcomes to support a valid non-inferiority test. Second, the study population was from two southern hemisphere countries on different continents, and was representative of individuals from various racial and ethnic groups. Limitations of the study include the fact that the study analyzed surrogate immunogenicity endpoints and did not evaluate direct protection against influenza infection or disease. However, further studies upon the implementation of this vaccine can provide evidence for effectiveness in the real world. Finally, most participants were enrolled in the Philippines, and therefore no direct comparative analyses were conducted on data from these two countries.

## 5. Conclusions

In conclusion, Sinovac QIV showed satisfactory immunogenicity results in individuals aged 3 years and older in Chile and the Philippines, and non-inferiority was shown compared to a licensed QIV (Vaxigrip Tetra^®^). The safety profile of Sinovac QIV was similar to that of Vaxigrip Tetra^®^.

## Figures and Tables

**Table 1:** Demographic and other characteristics (Full analysis set).

Indicators	Sinovac QIV Group (N = 1019)	Comparator QIV Group (N = 1020)
Gender, male, n (%)	410 (40.2)	432 (42.4)
Age (yr), Mean ± SD	31.3 ± 25.7	31.9 ± 25.9
Age strata, n (%)		
3–8 years	249 (24.4)	251 (24.6)
Vaccine primed	7 (0.7)	10 (1.0)
Vaccine unprimed	242 (23.7)	241 (23.6)
9–17 years	250 (24.5)	250 (24.5)
18–64 years	270 (26.5)	269 (26.4)
≥65 years	250 (24.5)	250 (24.5)
Height (cm), Mean ± SD	143.9 ± 22.7	144.0 ± 23.0
Weight (kg), Mean ± SD	47.5 ± 22.2	47.6 ± 22.5
Country n (%)		
Chile	166 (16.3)	166 (16.3)
The Philippines	853 (83.7)	854 (83.7)
Race n (%)		
Asian	852 (83.6)	854 (83.7)
White	166 (16.3)	166 (16.3)
Black or African American	1 (0.1)	0 (0.0)
Ethnicity n (%)		
Hispanic or Latino	160 (15.7)	165 (16.2)
Not Hispanic or Latino	859 (84.3)	855 (83.8)

Abbreviations: SD: Standard Deviation.

**Table 2:** Immunogenicity non-inferiority results of HI antibody SCR and GMT at 28 days after final vaccination (Per-protocol set).

Age	Strain	SCR Difference (Sinovac QIV-Comparator QIV)		Adjusted GMT Ratio (Sinovac QIV/Comparator QIV)	
		Rate (95% CI)	Non-Inferiority	Ratio (95% CI)	Non-Inferiority
All ages	A (H1N1)	9.6 (6.7, 12.5)	Yes	1.8 (1.6, 2.0)	Yes
	A (H3N2)	7.0 (3.5, 10.5)	Yes	1.4 (1.3, 1.6)	Yes
	B Victoria	2.4 (−0.03, 4.9)	Yes	1.3 (1.1, 1.4)	Yes
	B Yamagata	6.8 (3.0, 10.7)	Yes	1.2 (1.1, 1.2)	Yes
3–8 years	A (H1N1)	2.4 (−1.7, 6.6)	Yes	1.8 (1.5, 2.1)	Yes
	A (H3N2)	8.6 (2.7, 14.5)	Yes	1.5 (1.2, 1.8)	Yes
	B Victoria	0.4 (−0.4, 1.2)	Yes	1.2 (1.0, 1.5)	Yes
	B Yamagata	6.9 (0.7, 13.2)	Yes	1.0 (0.9, 1.2)	Yes
9–17 years	A (H1N1)	4.5 (−0.4, 9.4)	Yes	1.6 (1.3, 1.9)	Yes
	A (H3N2)	4.6 (−2.7, 11.9)	Yes	1.6 (1.4, 2.0)	Yes
	B Victoria	1.7 (−2.1, 5.4)	Yes	1.3 (1.1, 1.6)	Yes
	B Yamagata	−0.3 (−6.6, 6.0)	Yes	1.3 (1.1, 1.5)	Yes
18–64 years	A (H1N1)	14.6 (8.4, 20.9)	Yes	1.7 (1.3, 2.1)	Yes
	A (H3N2)	7.2 (0.4, 14.0)	Yes	1.3 (1.0, 1.6)	Yes
	B Victoria	8.3 (2.7, 13.9)	Yes	1.2 (1.0, 1.5)	Yes
	B Yamagata	10.1 (1.8, 18.4)	Yes	1.2 (1.1, 1.4)	Yes
**≥65 years**	A (H1N1)	16.5 (9.5, 23.5)	Yes	2.1 (1.7, 2.6)	Yes
	A (H3N2)	7.6 (0.0, 15.3)	Yes	1.4 (1.1, 1.7)	Yes
	B Victoria	−0.8 (−7.4, 5.8)	Yes	1.3 (1.1, 1.5)	Yes
	B Yamagata	10.4 (1.9, 19.0)	Yes	1.1 (1.0, 1.3)	Yes

Abbreviations: SCR, Seroconversion rate; GMT: geometric mean titer.

**Table 3:** Immunogenicity results at 28 days after the final vaccination (Per-protocol set).

		A(H1N1)			A (H3N2)			B Victoria			B Yamagata		
		Sinovac QIV	Comparator QIV	*p* Value	Sinovac QIV	Comparator QIV	*p* Value	Sinovac QIV	Comparator QIV	*p* Value	Sinovac QIV	Comparator QIV	*p* Value
All ages	**N**	**998**	**995**		**998**	**995**		**998**	**995**		**998**	**995**	
	SCR (%) (95%CI)	91.8 (89.9, 93.4)	82.2 (79.7, 84.5)	<0.0001	83.5 (81.0, 85.7)	76.5 (73.7, 79.1)	<0.0001	92.6 (90.8, 94.1)	90.2 (88.1, 91.9)	0.0530	77.1 (74.3, 79.6)	70.3 (67.3, 73.1)	0.0006
	SPR (%) (95%CI)	96.8 (95.5, 97.8)	93.2 (91.4, 94.7)	0.0002	99.4 (98.7, 99.8)	99.4 (98.7, 99.8)	0.9958	99.9 (99.4, 100.0)	98.8 (97.9, 99.4)	0.0022	99.9 (99.4, 100.0)	100.0 (99.6, 100.0)	1.0000
	GMT (95%CI)	293.7 (272.3,316.7)	166.8 (155.2, 179.4)	<0.0001	740.2 (684.5, 800.5)	519.3 (483.5, 557.8)	<0.0001	515.8 (479.5, 554.8)	412.1 (383.1, 443.2)	<0.0001	329.7 (313.0, 347.3)	285.3 (271.2, 300.0)	<0.0001
	GMI (95%CI)	15.7 (14.7, 16.7)	12.8 (12.0, 13.6)	<0.0001	5.7 (5.5, 5.9)	5.2 (5.0, 5.4)	0.0008	8.6 (8.2, 9.0)	8.0 (7.6, 8.4)	0.0523	4.9 (4.7, 5.0)	4.7 (4.5, 4.9)	0.1559
3–8 years	**N**	**243**	**244**		**243**	**244**		**243**	**244**		**243**	**244**	
	SCR (%) (95%CI)	95.5 (92.0, 97.7)	93.0 (89.1, 95.9)	0.2474	91.4 (87.1, 94.6)	82.8 (77.5, 87.3)	0.0048	100.0 (98.5, 100.0)	99.6 (97.7, 100.0)	1.0000	88.9 (84.3, 92.6)	82.0 (76.6, 86.6)	0.0304
	SPR (%) (95%CI)	99.2 (97.1, 99.9)	98.0 (95.3, 99.3)	0.4497	99.2 (97.1, 99.9)	100.0 (98.5, 100.0)	0.2485	100.0 (98.5, 100.0)	100.0 (98.5, 100.0)	NA	99.6 (97.7, 100.0)	100.0 (98.5, 100.0)	0.4990
	GMT (95%CI)	338.8 (298.3, 384.8)	189.2 (167.3, 213.9)	<0.0001	1518.9 (1310.5, 1760.6)	1043.2 (931.9, 1167.9)	<0.0001	948.7 (826.2, 1089.4)	787.5 (698.6, 887.7)	0.0460	268.1 (240.7, 298.8)	261.6 (235.5, 290.5)	0.7468
	GMI (95%CI)	17.4 (15.4, 19.7)	14.5 (13.0, 16.2)	0.0332	5.6 (5.2, 6.0)	4.8 (4.5, 5.2)	0.0027	11.4 (10.3, 12.5)	10.4 (9.4, 11.5)	0.2321	6.5 (6.0, 7.2)	6.1 (5.6, 6.7)	0.3379
9–17 years	**N**	**250**	**248**		**250**	**248**		**250**	**248**		**250**	**248**	
	SCR (%) (95%CI)	93.6 (89.8, 96.3)	89.1 (84.6, 92.7)	0.0747	80.0 (74.5, 84.8)	75.4 (69.6, 80.6)	0.2178	96.0 (92.8, 98.1)	94.4 (90.7, 96.9)	0.3914	84.8 (79.7, 89.0)	85.1 (80.0, 89.3)	0.9302
	SPR (%) (95%CI)	98.4 (96.0, 99.6)	98.4 (95.9, 99.6)	1.0000	100.0 (98.5, 100.0)	100.0 (98.5, 100.0)	NA	100.0 (98.5, 100.0)	98.4 (95.9, 99.6)	0.1300	100.0 (98.5, 100.0)	100.0 (98.5, 100.0)	NA
	GMT (95%CI)	444.3 (383.8, 514.4)	284.6 (248.3, 326.1)	<0.0001	984.7 (860.9, 1126.3)	605.2 (532.8, 687.4)	<0.0001	412.1 (356.4, 476.5)	309.5 (266.9, 358.7)	0.0067	418.6 (375.4, 466.8)	328.2 (295.6, 364.2)	0.0016
	GMI (95%CI)	20.8 (18.1, 23.8)	17.6 (15.4, 20.1)	0.0862	5.3 (4.9, 5.6)	5.0 (4.7, 5.4)	0.3014	9.7 (8.9, 10.7)	9.7 (8.9, 10.6)	0.9656	5.5 (5.1, 5.9)	5.4 (5.0, 5.8)	0.8351
18–64 years	**N**	**256**	**254**		**256**	**254**		**256**	**254**		**256**	**254**	
	SCR (%) (95%CI)	91.0 (86.8, 94.2)	76.4 (70.7, 81.5)	<0.0001	84.4 (79.3, 88.6)	77.2 (71.5, 82.2)	0.0388	92.2 (88.2, 95.2)	83.9 (78.8, 88.2)	0.0038	68.8 (62.7, 74.4)	58.7 (52.3, 64.8)	0.0178
	SPR (%) (95%CI)	94.1 (90.5, 96.7)	87.8 (83.1, 91.6)	0.0124	99.2 (97.2, 99.9)	98.4 (96.0, 99.6)	0.6742	99.6 (97.8, 100.0)	97.6 (94.9, 99.1)	0.1253	100.0 (98.6, 100.0)	100.0 (98.6, 100.0)	NA
	GMT (95%CI)	204.7 (175.2, 239.2)	123.8 (106.9, 143.4)	<0.0001	384.7 (332.3, 445.4)	302.2 (263.0, 347.2)	0.0191	316.6 (278.2, 360.3)	261.5 (226.8, 301.5)	0.0515	314.8 (287.6, 344.6)	260.1 (236.2, 286.4)	0.0046
	GMI (95%CI)	15.5 (13.7, 17.6)	12.1 (10.7, 13.6)	0.0049	6.4 (5.9, 7.0)	6.0 (5.5, 6.6)	0.3317	8.0 (7.3, 8.7)	6.9 (6.3, 7.5)	0.0202	4.0 (3.9, 4.1)	3.8 (3.7, 4.0)	0.0769
≥65 years	**N**	**249**	**249**		**249**	**249**		**249**	**249**		**249**	**249**	
	SCR (%) (95%CI)	87.2 (82.3, 91.0)	70.7 (64.6, 76.3)	<0.0001	78.3 (72.7, 83.3)	70.7 (64.6, 76.3)	0.0508	82.3 (77.0, 86.9)	83.1 (77.9, 87. 6)	0.8126	66.3 (60.0, 72.1)	55.8 (49.4, 62.1)	0.0169
	SPR (%) (95%CI)	95.6 (92.2, 97.8)	88.8 (84.2, 92.4)	0.0046	99.2 (97.1, 99.9)	99.2 (97.1, 99.9)	1.0000	100.0 (98.5, 100.0)	99.2 (97.1, 99.9)	0.4786	100.0 (98.5, 100.0)	100.0 (98.5, 100.0)	NA
	GMT (95%CI)	244.3 (209.9, 284.4)	117.5 (101.8, 135.6)	<0.0001	540.1 (469.7, 620.9)	391.0 (339.9, 449.8)	0.0014	588.7 (514.7, 673.4)	462.1 (405.1, 527.1)	0.0116	332.7 (300.6, 368.2)	296.8 (270.2, 326.2)	0.1060
	GMI (95%CI)	10.8 (9.5, 12.2)	8.8 (7.8, 9.9)	0.0167	5.5 (5.1, 5.9)	4.9 (4.6, 5.3)	0.0441	6.2 (5.7, 6.8)	6.0 (5.5, 6.5)	0.6231	4.0 (3.8, 4.1)	3.9 (3.7, 4.0)	0.3469

Abbreviations: SPR, Seroprotection rate; SCR, Seroconversion rate: GMT: geometric mean titer; GMI: geometric mean increase.

**Table 4:** The incidence of adverse reactions until 28 days after the final vaccination (Safety set).

Adverse Reactions	Sinovac QIV Group	Comparator QIV Group	*p*-Value
	(N = 1017)	(N = 1022)	
Overall adverse reactions	259(25.5)	276(27.0)	0.4297
Grade 1	227(22.3)	235(23.0)	0.7164
Grade 2	83(8.2)	92(9.0)	0.4980
Grade 3	10(1.0)	7(0.7)	0.4588
Solicited adverse reactions	252(24.8)	263(25.7)	0.6197
Local	166(16.3)	175(17.1)	0.6281
Pain	157(15.4)	165(16.1)	0.6615
Pruritus	12(1.2)	29(2.8)	0.0077
Swelling	15(1.5)	13(1.3)	0.6938
Erythema	10(1.0)	14(1.4)	0.4184
Induration	8(0.8)	11(1.1)	0.4960
Rash	1(0.1)	2(0.2)	1.0000
Systemic	152(14.9)	163(15.9)	0.5309
Headache	77(7.6)	81(7.9)	0.7648
Fever	51(5.0)	57(5.6)	0.5707
Fatigue	50(4.9)	51(5.0)	0.9388
Cough	38(3.7)	45(4.4)	0.4463
Muscle pain	30(2.9)	35(3.4)	0.5417
Diarrhea	20(2.0)	17(1.7)	0.6081
Nausea	23(2.3)	13(1.3)	0.0898
Anorexia	12(1.2)	18(1.8)	0.2757
Vomiting	4(0.4)	9(0.9)	0.2653
Skin and mucosa abnormality	8(0.8)	3(0.3)	0.1440
Acute allergic reaction	1(0.1)	3(0.3)	0.6246
Unsolicited adverse reactions	22(2.2)	30(2.9)	0.2688

Data is presented as n (%).

## Data Availability

Data supporting findings of this study can be found in the article and Supplementary Materials. Original data in a de-identified format and study protocols are available from corresponding authors, upon reasonable request for research purposes. A materials transfer and/or data access agreement with the sponsor will be required for accessing shared data.

## Supplementary Materials

The following supporting information can be downloaded at: https://www.mdpi.com/article/10.3390/vaccines12080892/s1, Table S1: Baseline antibody level (per-protocol set); Table S2: Immunogenicity results in participants aged 18-64 years in Chile and the Philippines (per-protocol set); Table S3: Study site, IRB/IEC institution and approval Number.
