# Supplementary material for: Immunogenicity and Safety of a Quadrivalent Influenza Vaccine in Population Aged 3 Years and Older in Chile and the Philippines: A Phase 3, Non-Inferiority, Double-Blind, Randomized Controlled Clinical Trial
Source: Vaccines (Basel). 2024 Aug 7;12(8):892. doi: 10.3390/vaccines12080892 (PMC11360333; doi:10.3390/vaccines12080892)
Supplement: Supplementary file 1 [file vaccines-12-00892-s001.zip › vaccines-3092985-supplementary.pdf]

Supplement Table S1. Baseline antibody level (per-protocol set).

		A(H1N1)			A (H3N2)			B Victoria			B Yamagata		
		**Sinovac QIV**	**Comparator QIV**	**P value**	**Sinovac QIV**	**Comparator QIV**	**P value**	**Sinovac QIV**	**Comparator QIV**	**P value**	**Sinovac QIV**	**Comparator QIV**	**P value**
**All ages**	**N**	**998**	**995**		**998**	**995**		**998**	**995**		**998**	**995**	
	SPR (%)	18.4	16.5	0.2505	73.4	74.7	0.4995	43.1	43.3	0.9173	76.9	76.0	0.6459
	(95%CI)	(16.1,21.0)	(14.2,18.9)		(70.5, 76.1)	(71.9, 77.4)		(40.0, 46.2)	(40.2, 46.5)		(74.1, 79.4)	(73.2, 78.6)	
	GMT	10.6	10.0	0.1048	64.8	64.5	0.9891	25.3	24.9	0.9136	50.9	49.9	0.5897
	(95%CI)	(10.0,11.3)	(9.4,10.7)		(59.6,70.4)	(59.6,69.8)		(23.6,27.2)	(23.2,26.7)		(48.0,54.1)	(47.2,52.8)	
**3-8 years**	**N**	**243**	**244**		**243**	**244**		**243**	**244**		**243**	**244**	
	SPR (%)	18.5	14.8	0.2647	86.0	91.4	0.0605	44.4	44.7	0.9597	58.9	59.0	0.9698
	(95%CI)	(13.8,24.0)	(10.6,19.8)		(81.0,90.1)	(87.1,94.6)		(38.1,50.9)	(38.3,51.2)		(52.4,65.1)	(52.6,65.3)	
	GMT	10.4	9.1	0.2142	96.9	115.7	0.1765	22.6	23.9	0.6743	27.3	29.7	0.4948
	(95%CI)	(9.1,11.9)	(8.0,10.2)		(82.6,113.6)	(100.0,133.9)		(19.6,25.9)	(20.8,27.4)		(24.2,30.8)	(26.4,33.4)	
**9-17 years**	**N**	**250**	**248**		**250**	**248**		**250**	**248**		**250**	**248**	
	SPR (%)	17.2	15.3	0.5703	80.4	77.8	0.4793	31.2	21.8	0.0172	69.2	68.6	0.8752
	(95%CI)	(12.7,22.5)	(11.1,20.4)		(74.9,85.1)	(72.1,82.8)		(25.5,37.3)	(16.8,27.4)		(63.1,74.9)	(62.4, 74.3)	
	GMT	10.2	9.8	0.7162	89.4	76.1	0.2321	18.1	15.5	0.0555	44.4	38.4	0.0719
	(95%CI)	(8.9,11.6)	(8.6,11.1)		(76.0,105.2)	(64.9,89.2)		(16.0,20.5)	(13.8,17.5)		(39.7,49.7)	(34.7,42.4)	
**18-64 years**	**N**	**256**	**254**		**256**	**254**		**256**	**254**		**256**	**254**	
	SPR (%)	11.7	11.0	0.8047	57.4	56.7	0.8679	35.2	41.7	0.1269	90.6	89.0	0.5384
	(95%CI)	(8.1,16.3)	(7.5,15.5)		(51.1,63.6)	(50.4,62.9)		(29.3,41.4)	(35.6,48.1)		(86.4,93.9)	(84.5,92.6)	
	GMT	8.6	8.7	0.9072	36.2	34.7	0.7824	20.8	24.1	0.1169	70.3	69.0	0.7494
	(95%CI)	(7.8,9.6)	(7.8,9.7)		(30.9,42.4)	(29.9,40.2)		(18.5,23.5)	(21.1,27.4)		(64.2,76.8)	(63.2,75.4)	
**≥65 years**	**N**	**249**	**249**		**249**	**249**		**249**	**249**		**249**	**249**	
	SPR (%)	26.5	24.9	0.6817	70.3	73.5	0.4252	61.9	65.1	0.5381	88.0	86.8	0.6859
	(95%CI)	(21.1,32. 5)	(19.7,30.8)		(64.2,75.9)	(67.6,78.9)		(55.5,67.9)	(58.8,71.0)		(83.3,91.7)	(81.9,90.7)	
	GMT	14.0	13.0	0.1512	57.6	58.1	0.8601	48.3	43.0	0.4630	77.2	77.6	0.6730
	(95%CI)	(12.3,15.9)	(11.2,15.1)		(49.2,67.4)	(50.2,67.2)		(41.4,56.5)	(37.3,49.6)		(69.1,86.1)	(69.8,86.2)	

Supplementary Table S2. Immunogenicity results in participants aged 18-64 years in Chile and the Philippines (per-protocol set)*.

			A(H1N1)			A (H3N2)			B Victoria			B Yamagata		
			**Sinovac QIV**	**Control QIV**	**P value***	**Sinovac QIV**	**Control QIV**	**P value***	**Sinovac QIV**	**Control QIV**	**P value***	**Sinovac QIV**	**Control QIV**	**P value***
**Pre**	**CL**	**N**	113	111		113	111		113	111		113	111	
		SPR (%) (95%CI)	13.27 (7.62, 20.95)	13.51 (7.77, 21.31)	0.9581	38.94 (29.91, 48.56)	37.84 (28.80, 47.54)	0.8656	47.79 (38.30, 57.39)	54.95 (45.22, 64.41)	0.2832	99.12 (95.17, 99.98)	97.30 (92.30, 99.44)	0.3044
		GMT (95%CI)	9.82 (8.26,11.66)	10.13 (8.50,12.06)	0.7966	24.19 (19.05,30.71)	22.66 (18.08,28.41)	0.8026	28.90 (23.95,34.86)	34.01 (28.09,41.16)	0.2059	72.97 (65.37,81.44)	79.50 (71.39,88.54)	0.1587
	**PH**	**N**	143	143		143	143		143	143		143	143	
		SPR (%) (95%CI)	10.49 (5.99, 16.71)	9.09 (4.93, 15.04)	0.6907	72.03 (63.91, 79.21)	71.33 (63.18, 78.58)	0.8956	25.17 (18.30, 33.11)	31.47 (23.97, 39.76)	0.2375	83.92 (76.85, 89.52)	82.52 (75.28, 88.36)	0.7517
		GMT (95%CI)	7.77 (6.84,8.83)	7.77 (6.81,8.87)	0.9602	49.75 (40.79,60.68)	48.32 (40.36,57.85)	0.8813	16.08 (13.88,18.63)	18.42 (15.58,21.77)	0.2929	68.17 (59.52,78.09)	61.88 (54.28,70.53)	0.1746
**Post**	**CL**	N	113	111		113	111		113	111		113	111	
		SCR (%) (95%CI)	84.96 (77.01, 90.99)	64.86 (55.23, 73.69)	**0.0005**	82.30 (74.00, 88.84)	72.07 (62.76, 80.17)	0.0680	87.61 (80.09, 93.06)	80.18 (71.54, 87.14)	0.1300	59.29 (49.65, 68.44)	47.75 (38.18, 57.44)	0.0832
		SPR (%) (95%CI)	89.38 (82.18, 94.39)	81.98 (73.55, 88.63)	0.1136	98.23 (93.75, 99.78)	96.40 (91.03, 99.01)	0.3954	100.00 (96.79, 100.00)	100.00 (96.73, 100.00)	NA	100.00 (96.79, 100.00)	100.00 (96.73, 100.00)	NA
		GMT (95%CI)	113.48 (93.48,137.77)	80.00 (64.55,99.15)	**0.0132**	250.37 (200.59,312.52)	199.09 (162.06,244.57)	0.1493	320.00 (269.03,380.63)	289.57 (239.46,350.18)	0.2206	266.21 (234.49,302.23)	247.72 (216.69,283.19)	0.3295
		GMI (95%CI)	11.43 (9.37,13.93)	7.99 (6.54,9.77)	0.1803	10.59 (8.66,12.95)	8.58 (7.00,10.52)	0.4392	10.48 (8.84,12.43)	9.01 (7.58,10.70)	0.5570	3.53 (3.11,4.01)	3.22 (2.84,3.67)	0.5596
	**PH**	N	143	143		143	143		143	143		143	143	
		SCR (%) (95%CI)	95.80 (91.09, 98.44)	85.31 (78.43, 90.67)	**0.0024**	86.01 (79.23, 91.24)	81.12 (73.73, 87.17)	0.2640	95.80 (91.09, 98.44)	86.71 (80.03, 91.81)	**0.0065**	76.22 (68.39, 82.94)	67.13 (58.79, 74.75)	0.0880
		SPR (%) (95%CI)	97.90 (93.99, 99.57)	92.31 (86.65, 96.10)	**0.0283**	100.00 (97.45, 100.00)	100.00 (97.45, 100.00)	NA	99.30 (96.17, 99.98)	95.80 (91.09, 98.44)	0.0557	100.00 (97.45, 100.00)	100.00 (97.45, 100.00)	NA
		GMT (95%CI)	326.26 (265.47,400.98)	173.74 (144.01,209.61)	**<0.0001**	540.13 (450.69,647.32)	417.76 (350.82,497.48)	**0.0412**	313.86 (259.45,379.66)	241.58 (196.10,297.60)	**0.0229**	359.48 (317.12,407.49)	270.07 (235.06,310.29)	**0.0045**
		GMI (95%CI)	41.98 (34.49,51.10)	22.35 (18.36,27.21)	0.0544	10.97 (9.27,12.98)	8.56 (7.23,10.13)	0.2923	18.71 (15.47,22.63)	13.68 (11.31,16.55)	0.2555	5.41 (4.81,6.09)	4.25 (3.78,4.78)	0.1329

Abbreviations: Abbreviations: SPR, Seroprotection rate; SCR, Seroconversion rate; GMT: geometric mean titer; GMI: geometric mean increase; CL, Chile; PH, the Philippines

*A total of 297 participants from Chile were included in PPS, including 224 aged 18-64 years, 5 aged 3-8 years, 36 aged 9-17 years, 32 aged 65 years and older. Analysis was conducted in 18-64 year old group, considering insufficient sample size in other age strata.

Supplementary Table S3. Study site, IRB/IEC institution and approval Number.

**Site No.**	**Study center**	IRB/IEC institution	Approval Number	Approval date
PH01	Las Pinas Doctors Hospital, Philippines	UNIVERSITY OF THE EAST RAMON MAGSAYSAY MEMORIAL MEDICAL CENTER, Ethics Review for Health Science of Research Institute for Health	1425/P/2022/235	21-Feb-2023
PH02	University Of the Philippines - Philippines General Hospital, Philippines	University of the Philippines Manila Research Ethics Board	UPMREB 2022-0614-01	21-Feb-2023
PH03	Tropical Disease Foundation Inc., Philippines	Makati Medical Center Institutional Review Board	MMCIRB 2022-172	05-Feb-2023
PH04	San Juan De Dios Hospital Philippines	San Juan De Dios Hospital Institution Review Board	SJIRB-2022-0045-EMED	23-Feb-2023
CL01	Centro Marcoleta, Red de Salud UC Christus, Santiago, Chile	Institutional Scientific Ethical Committeeof Health Sciences of the Pontificia Universidad Católica de Chile	220311002	25-Nov-2022
CL04	Centro CIMER/Centro de Investigaciones Médicas de Enfermedades Respiratorias, Santiago, Chile			
CL05	Centro Clínica Alemana, Santiago, Chil			
CL07	Centro Hospital de Puerto Montt, Puerto Montt, Chile			
CL08	Centro de Salud Universidad San Sebastián (USS), Chile			
